# The Application of NHEJ-CRISPR/Cas9 and Cre-Lox System in the Generation of Bivalent Duck Enteritis Virus Vaccine against Avian Influenza Virus

**DOI:** 10.3390/v10020081

**Published:** 2018-02-13

**Authors:** Pengxiang Chang, Yongxiu Yao, Na Tang, Jean-Remy Sadeyen, Joshua Sealy, Anabel Clements, Sushant Bhat, Muhammad Munir, Juliet E. Bryant, Munir Iqbal

**Affiliations:** 1 The Pirbright Institute, Pirbright, Woking GU24 0NF, UK; pengxiang.chang@pirbright.ac.uk (P.C.); yongxiu.yao@pirbright.ac.uk (Y.Y.); jean-remy.sadeyen@pirbright.ac.uk (J.-R.S.); joshua.sealy@pirbright.ac.uk (J.S.); anabel.clements@pirbright.ac.uk (A.C.); sushant.bhat@pirbright.ac.uk (S.B.); 2 Shandong Binzhou Animal Science and Veterinary Medicine Academy, Binzhou 256600, Shandong, China; na.tang@pirbright.ac.uk; 3 Division of Biomedical and Life Sciences, Faculty of Health and Medicine, Furness Building, Lancaster University, Bailrigg, Lancaster LA1 4YG, UK; Drmunir.muhammad@gmail.com; 4 Laboratory of Emerging Pathogens, Fondation Mérieux, 69002 Lyon, France; jebryant13@gmail.com

**Keywords:** duck enteritis virus, CRISPR/Cas9, NHEJ, influenza, Cre, Lox

## Abstract

Duck-targeted vaccines to protect against avian influenza are critically needed to aid in influenza disease control efforts in regions where ducks are endemic for highly pathogenic avian influenza (HPAI). Duck enteritis virus (DEV) is a promising candidate viral vector for development of vaccines targeting ducks, owing to its large genome and narrow host range. The clustered regularly interspaced palindromic repeats (CRISPR)/Cas9 system is a versatile gene-editing tool that has proven beneficial for gene modification and construction of recombinant DNA viral vectored vaccines. Currently, there are two commonly used methods for gene insertion: non-homologous end-joining (NHEJ) and homology-directed repair (HDR). Owing to its advantages in efficiency and independence from molecular requirements of the homologous arms, we utilized NHEJ-dependent CRISPR/Cas9 to insert the influenza hemagglutinin (HA) antigen expression cassette into the DEV genome. The insert was initially tagged with reporter green fluorescence protein (GFP), and a Cre-Lox system was later used to remove the *GFP* gene insert. Furthermore, a universal donor plasmid system was established by introducing double bait sequences that were independent of the viral genome. In summary, we provide proof of principle for generating recombinant DEV viral vectored vaccines against the influenza virus using an integrated NHEJ-CRISPR/Cas9 and Cre-Lox system.

## 1. Introduction

Duck enteritis virus (DEV) is an alpha-herpesvirus that infects ducks, geese and swans [1]. It causes acute contagious diseases in susceptible birds with mortality rates that can reach 100% in ducks, and is therefore a significant economic concern [1]. The viral genome comprises a double-stranded DNA of about 160 kilobase pairs, and comprises a number of unique long (UL) and unique short (US) sequence formats. Live attenuated DEV vaccines are widely used for reduction of diseases impact in ducks [1]. Due to its large genome and narrow host range, DEV has been exploited as a vector for development of recombinant multivalent vaccines [2,3]. Virus vector vaccines have advantages over inactivated vaccines through induction of both cellular and humoral responses, and enabling differentiation between infected and vaccinated animals (DIVA) approaches [4].

Avian influenza viruses (AIV) are enveloped and carry a segmented and negative-strand RNA genome characterized by very high evolutionary rates that facilitate the process of antigenic drift and immune escape. Aquatic birds form the enzootic reservoir for the majority of subtypes of influenza A viruses [5]. Domestic duck populations in southeast Asia are considered to play a key role in maintenance of highly pathogenic avian influenza (HPAI) subtype H5N1 viruses [6]. Ducks can frequently sustain HPAI infection without any overt signs of disease, thus enabling “silent” transmission cycles and continued opportunities for “spillover” transmission to in-contact chickens, causing outbreaks that are often extremely lethal and difficult to contain [7]. Thus, vaccination to protect ducks against H5 HPAI is warranted to reduce production losses to duck farmers, to safeguard against transmission to other poultry species, and to mitigate the risks for zoonotic emergence.

Several genome modification methods have been adopted in the past to produce duck enteritis vector vaccines, such as homologous recombination, bacterial artificial chromosome (BAC), and fosmid system construction [2,8,9]. However, these methods are generally time-consuming and labor-intensive. Clustered regularly interspaced palindromic repeats (CRISPR)/associated (Cas9) is a gene-editing technology that has gained popularity in recent years for its versatility and specificity. In this system, a single guide RNA (sgRNA) recognizes a 20 nucleotide target sequence adjacent to a 5′ NGG 3′ protospacer adjacent motif (PAM), and Cas9 introduces a double strand break (DSB) in this target sequence. The DSBs can then be repaired by either the error-prone non-homologous end-joining (NHEJ) or the high-fidelity homology-directed repair (HDR) pathway [10]. Extensive research has demonstrated the value of the HDR-CRISPR/Cas9 system for vaccine development [3,11,12], whereas the alternative approach utilizing the NHEJ-CRISPR/Cas9 system is less well established, despite potential higher insertion efficiency in comparison to the HDR method.

The Cre-Lox system is a site-specific recombination system that has been used to excise BAC [13]. The Cre recombinase enzyme, originally derived from the P1 bacteriophage, can recognize specific 34 base-pair DNA sequences called Lox sites and the DNA between Lox sites can be excised [14].

In this study, we employed the NHEJ-CRISPR/Cas9 system for DEV-AIV bivalent vaccine development. We introduced a GFP expression cassette into the DEV genome as an indicator to confirm the foreign gene insertion and expression, which was later removed using the Cre-Lox system. We show that NHEJ-CRISPR/Cas9 together with Cre-Lox is an efficient method for rapid generation of a recombinant DEV-AIV vaccine.

## 2. Materials and Methods

### 2.1. Viruses, Cells and Transfection

We obtained a prototype strain of duck enteritis virus from LGC Standards (England, UK) (ATCC^®^ VR-684™). The virus was propagated in primary chick embryo fibroblasts (CEF) prepared from 10-day-old specific-pathogen-free embryonated chicken eggs, and virus stocks were kept at −80 °C. Cells were maintained with Dulbecco’s Modified Eagle’s medium (DMEM) (Gibco, Life Technologies Ltd., Paisley, UK), supplemented with 10% fetal calf serum (FCS) (Gibco), 100 U/mL penicillin, and 100 μg/mL streptomycin (Gibco) at 37 °C under a 5% CO_2_ atmosphere. CEF cells were transfected with plasmids using TransIT-X2^®^ (Mirus, Cambridge Bioscience, Cambridge, UK) according to the manufacture’s protocol.

### 2.2. Virus Infection and Titration

CEF cells were washed once with phosphate-buffered saline (PBS) before being infection with the DEV virus. The inoculum was removed at 2 h post-infection and replenished with either fresh medium or 2% Minimum Essential Medium (MEM)-agarose overlay. The MEM-agarose overlay medium contains MEM (Sigma, St Louis, MO, USA), 2% agarose (Thermo Fisher Scientific, Waltham, MA, USA), 100 units/mL penicillin, 100 µg/mL streptomycin, 2 mM l-glutamine (Sigma), 0.3% bovine serum albumin (BSA) (Sigma), 15 mM 4-(2-hydroxyethyl)-1-piperazineethanesulfonic acid (HEPES) (Sigma), 0.22% sodium bicarbonate (Sigma) and 0.01% Diethylaminoethyl (DEAE)-Dextran (Sigma). For the virus plaque titration, infected cells were incubated at 37 °C under a 5% CO_2_ atmosphere for 5 days before being fixed with 1% crystal violet (Sigma) in 20% ethanol for plaque counting and plaque size measurement.

### 2.3. Multi-Step Growth Curve

CEF cells were infected with DEV at multiplicity of infection (MOI) 0.01. The supernatant and cells were harvested at 6 h, 12 h, 24 h, 48 h and 72 h post-infection. The harvested virus was kept at −80 °C until further analysis.

### 2.4. Immunochemistry

CEF cells were infected with DEV at MOI 0.01 for 48 h and then fixed in acetone:methanol (1/1) for 10 min, followed by incubation in blocking buffer (5% FCS in PBS) for 10 min. The expression of H5 hemagglutinin (HA) antigen in DEV-AIV vaccine infected cells was visualized by incubating cells with AIV H5 HA-specific antibody (mouse monoclonal) diluted in blocking buffer (1 in 1000 dilution) for 1 h at room temperature. Cells were subsequently rinsed with PBS and probed with horseradish peroxidase-labeled rabbit anti-mouse immunoglobulins (DAKO, Agilent Technologies, Santa Clara, CA, USA) for 40 min. After gentle rinsing with PBS, cells were stained with 3,3′-diaminobenzidine (DAB) substrate-chromogen solution (DAKO) for 7 min. The stained cell images were taken using Leica TCS SP5 confocal laser scanning microscope (Leica, Wetzlar, Germany).

### 2.5. Western Blot

DEV infected-CEF cells were lysed using Radioimmunoprecipitation assay (RIPA) Lysis and Extraction Buffer (Life Technologies Ltd, Paisley, UK). HA and alpha-tubulin proteins were detected via Western blot using mouse monoclonal antibody for influenza virus H5 HA (1 in 2000 dilution) and rabbit polyclonal anti-alpha tubulin (Abcam, Cambridge, UK) antibody (1 in 6000 dilution) with corresponding secondary anti-mouse or anti-rabbit IgG antibodies (both were 1 in 10,000 dilution) labeled with florescent dyes IRDye 800CW or IRDye 680RD (Li-COR, Lincoln, NE, USA) respectively and visualized using the Odyssey CLx (Li-COR).

### 2.6. DEV Genome Extraction and High-Resolution Melting (HRM)

CEF cells were transfected with 1 µg of sgRNA per well of a 12-well plate before infection with DEV at MOI 1.0. The DEV infected cells were harvested at 48 h post infection and lysed in 1× squishing buffer (10 mM Tris-HCl, pH 8, 1 mM EDTA, 25 mM NaCl, and 200 µg/mL Proteinase K) at 65 °C for 30 min. Primers for HRM analysis were designed using Primer Express 3 (Thermo Fisher Scientific). The PCR was performed in a 7500 fast real-time PCR machine (Applied Biosystems, Foster City, CA, USA) and analysed using Applied Biosystems™ HRM Software v3.0 according to the manufacturer’s instructions.

### 2.7. Construction of sgRNAs and Donor Plasmids

The single nucleotide guide RNA (sgRNA) was designed using CRISPR design tool (http://crispr.mit.edu/, Feng Zhang’s Lab). The universal bait sequence from copGFP was adapted from published data [15]. The DNA oligo of sgRNA was synthesized (Sigma) and cloned into plasmid pX459-v2 (Addgene, Cambridge, Massachusetts, USA) using BbsI cloning sites. DNA containing two copies of bait sequence, two copies of Lox site, PacI enzyme site and BsmBI enzyme site was synthesized (IDT, Leuven, Belgium) and further cloned into plasmid pExpreS2-v1 (ExpreS2ion Biotechnologies, Hørsholm, Demark). The resultant plasmid was designated pExpreS2-v1-SgU-Lox. The GFP expression cassette was amplified from plasmid pEGFP-N1 (Addgene) and subsequently cloned into PacI site of pExpreS2-v1-SgU-Lox to construct pExpreS2-v1-SgU-Lox-GFP. The HA expression cassette was amplified from pGEMT-H5N8-HA (A/duck/England/36254/14) plasmid and further cloned into BsmBI site of pExpreS2-v1-SgU-Lox-GFP, the resulting plasmid was termed pExpreS2-v1-SgU-Lox-GFP-HA.

### 2.8. NHEJ-CRISPR/Cas9-Mediated Gene Insertion

CEF cells were transfected with 0.3 µg sg2, 0.3 µg sgU and 0.6 µg donor plasmids per well of a 12-well plate before infection with DEV at different MOI. Virus was harvested at 48 h post infection and subjected to the plaque purification.

### 2.9. Cre Enzyme Treatment

The GFP expression cassette was excised by Cre recombinase. Cells were transfected with Cre recombinase plasmid and then infected with DEV at MOI 0.01 or 0.0025 at 24 h post-transfection. The supernatants were harvest at 48 h post-infection and kept at −80 °C until further analysis.

### 2.10. Statistical Analysis

Statistical analysis was performed using GraphPad Prism 6 (GraphPad Software, La Jolla, CA, USA). Paired student *t*-test and one-way ANOVA were used to test differences between different groups. *p* values < 0.05 were considered significant.

## 3. Results

### 3.1. Optimization of NHEJ-CRISPR/Cas9 System for Gene Knock-In

The intergenic region between DEV UL26 and UL27 was selected for insertion as previous research showed this site is compatible with foreign gene insertion [13]. High Resolution Melting (HRM) analysis is a rapid and sensitive method to identify variations in nucleic acid sequences [16]. This method was applied to select the most efficient sgRNA in insertion and deletion (indel) generation. In total, 3 sgRNAs against DEV were designed and cloned into px459-v2 plasmid (Table 1).

The CEF cells were transfected with sgRNA plasmids followed by infection with DEV virus at MOI 1.0. 48 h post-infection, genomic DNA from infected cells was extracted and subjected to HRM analysis. Primers to generate amplicons of ~200 bp traversing across the CRISPR target site are shown in Table 2.

**Figure 1:**
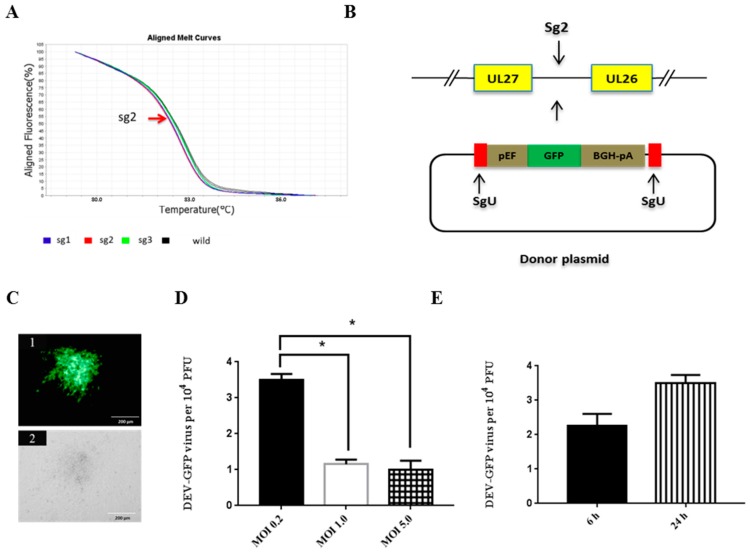
Optimization of gene knock-in via CRISPR/Cas9-induced NHEJ repair. (**A**) HRM analysis to select the most efficient sgRNA; (**B**) schematic presentation of insertion of GFP expression cassette between DEV UL26 and UL27; (**C**) DEV-GFP plaque under UV excitation (1) or phase contrast (2); (**D**) the efficiency of GFP cassette knock-in with different DEV infection dose; (**E**) the efficiency of GFP cassette knock-in with different infection time post transfection. Error bar = standard error of mean. * *p* < 0.05.

Based on the shift of the melting curve between wildtype and sgRNAs, sg2 sequence was selected as the most efficient sgRNA sequence for CRISPR/Cas9-based system for the production of recombinant DEV vaccine (Figure 1A).

To investigate the NHEJ-CRISPR/Cas9 mediated knock-in of foreign gene expression cassettes into the DEV genome, a GFP reporter gene was selected as a model. Two bait sequences derived from copGFP protein were introduced to 3′ and 5′ of the GFP expression cassette. sgU (adapted from published paper is listed in Table 1 [15]) targeting the bait sequence was introduced to cut the donor plasmid and release the GFP cassette segment (Figure 1B). CEF cells were transfected with sg2, sgU and donor plasmids and infected with DEV at MOI 1.0. The supernatant and cells were harvested and used to infect CEF cells. GFP positive plaque indicated successful insertion of GFP cassette into the DEV genome (Figure 1C). This virus is termed DEV-GFP. Bi et al. showed that the efficiency of generation indel by CRISPR/Cas9 is both dose- and time-post-transfection-dependent [12]. To determine the optimal virus dose for gene knock-in for DEV, cells were transfected with sg2, sgU and GFP donor plasmids and then infected with DEV at MOI 0.2, 1.0 and 5.0. The MOI 0.2 yielded the most efficient GFP knock-in as evidenced by GFP positive colonies (Figure 1D). Efficiency was reduced when higher infection doses of DEV virus (MOI 1.0 and MOI 5.0) were applied. To determine the optimal time for infection, CEF cells were transfected with sg2, sgU and GFP donor plasmids and then infected with DEV at MOI 0.2 at 6 or 24 h post-transfection, respectively. A slightly higher efficiency of GFP insertion was achieved at 24 h post-transfection in comparison to 6 h post-transfection (Figure 1E).

### 3.2. NHEJ-CRISPR/Cas9 Based Knock-In Is Non-Directional and the Unintended Indel Is Minimal

**Figure 2:**
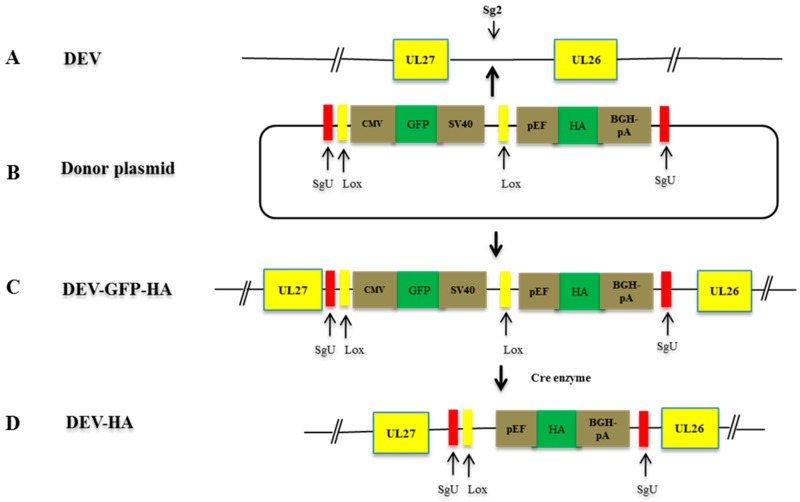
Schematic presentation of the application of NHEJ-CRISPR/Cas9 and Cre-Lox systems in construction of recombinant DEV-HA. (**A**) The live attenuated DEV virus genome with sg2 targeting the intergenic region between viral gene UL26 and UL27; (**B**) HA expression donor plasmid with GFP expression cassette flanked with Lox site. Bait sequence of SgU was introduced to both side of the insertion segment; (**C**) the recombinant DEV virus expressing HA and the reporter GFP; (**D**) the vaccine candidate with the GFP reporter expression cassette removed by recombinant Cre enzyme.

Given that we had demonstrated efficient GFP knock-in using NHEJ-CRISPR/Cas9 system, we proceeded to knock in an influenza H5 HA expression cassette into the DEV genome for the development of the bivalent vaccine against duck viral enteritis and avian influenza. To achieve this, a GFP reporter flanked with Lox sites was introduced into the HA donor plasmid as an indicator for insertion. The GFP cassette would be later removed by Cre recombinase enzyme (Figure 2).

**Figure 3:**
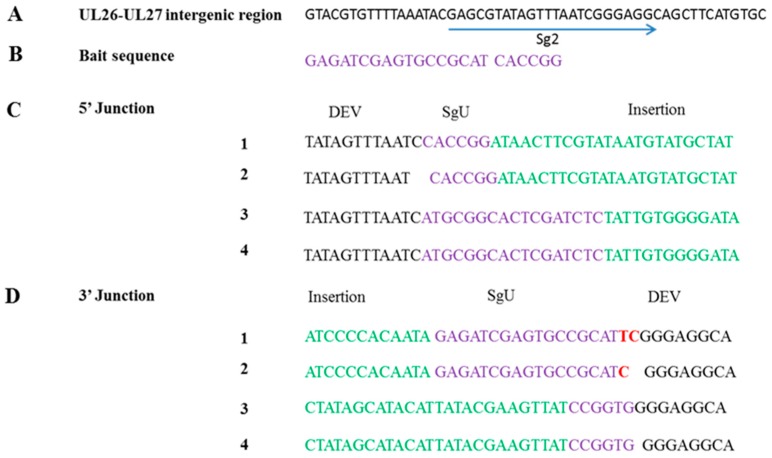
NHEJ-CRISPR/Cas9-based knock-in is non-directional and the unintended indels are minimal. (**A**) The schematic of sg2 target site in the intergenic region between DEV UL26 and UL27; (**B**) the target sequence of sgU; (**C**) the sequencing results of 5′ junction of 4 clones of DEV-GFP-HA; (**D**) the sequencing results of 3′ junction of 4 clones of DEV-GFP-HA. The DEV genome is colored in black; the sgU target sequence is colored purple; the insertion sequence is colored green; and the indel is colored in red.

CEF cells were transfected with HA donor plasmid (pExpreS2-v1-SgU-Lox-GFP-HA) and guide RNA plasmid (SgU, Sg2) and infected with DEV MOI 0.2 at 24 h post-transfection. Virus was harvested at 48 h post-infection and subjected to plaque purification, and the resultant recombinant termed DEV-GFP-HA. After 3 rounds of plaque purification, the genomic DNA of each virus colony was extracted and amplified using 5′ and 3′ junction specific primers (Table 2). PCR products were sequenced using commercial supplier (Source BioScience, Nottingham, UK). Sequencing results revealed that insertion occurred in both sense (colony 1 and 2) and anti-sense (colony 3 and 4) directions (Figure 3). For colony 1, two nucleotides thymine (T) and cytosine (C) were introduced at the 3′ junction. There was 1 nucleotide C deletion and a nucleotide C insertion at 5′ junction and 3′ junction respectively for virus colony 2. There were no indels for colony 3 or 4. The indel did not affect the open reading frame of the inserted HA gene expression cassette.

### 3.3. The Excision of the GFP Cassette from DEV-GFP-HA Is Virus Dose Dependent

**Figure 4:**
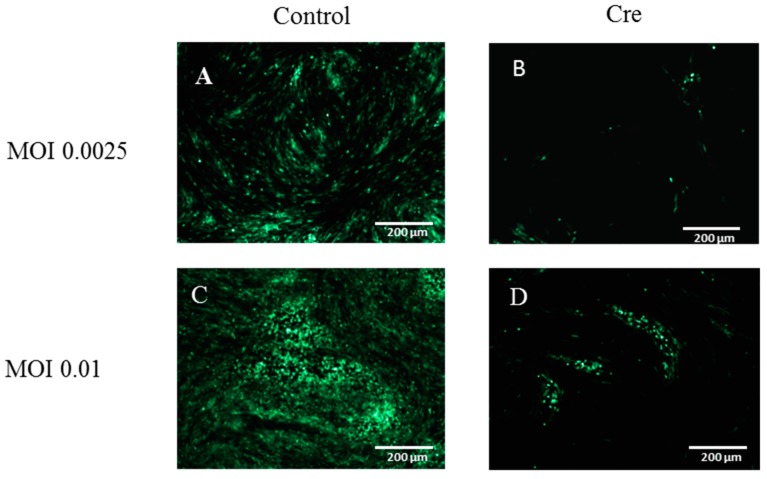

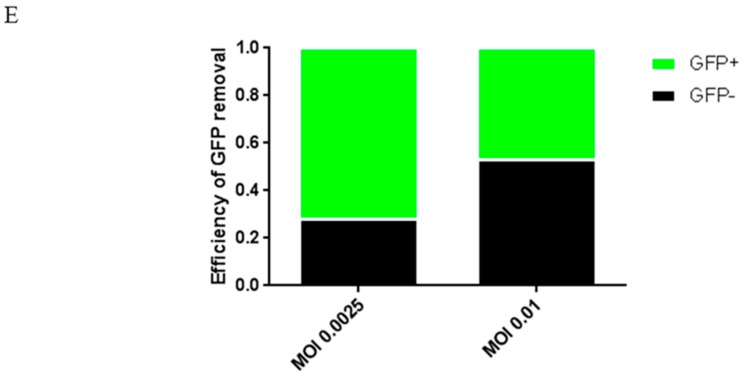
Optimization of GFP reporter removal via Cre-Lox system. CEF cells were transfected with Cre or control plasmids. At 24 h post transfection, the CEF cells were infected with DEV-GFP-HA at MOI 0.0025 or 0.01 respectively. Images were taken at 48 h post infection. (**A**) Empty vector with 0.0025 MOI infection; (**B**) Cre plasmid with 0.0025 MOI infection; (**C**) empty vector with MOI 0.01 infection; (**D**) Cre plasmid with 0.01 MOI infection; (**E**) the efficiency of GFP reporter removal with different infection DEV dose. The virus was harvested at 48 h post infection and was used to infect CEF cells; the percentage of GFP positive and negative plaques was calculated.

Cre-Lox has previously been used to remove the BAC sequence from recombinant duck enteritis virus [8,17]. However, the optimal condition for the Cre-Lox mediated excision has not previously been determined. In order to remove the GFP indicator, CEF cells were transfected with pcDNA3-Cre plasmid (Figure 4B,D) or the control plasmid (Figure 4A,C). 24 h post transfection, CEF cells were infected with DEV-GFP-HA virus at either MOI 0.0025 (Figure 4A,B) or MOI 0.01 (Figure 4C,D). 48 h post infection, virus was harvested and used to infect CEF cells. The ratio of GFP positive/negative colonies was calculated, which showed greater than 50% of GFP cassette was removed from the DEV-GFP-HA virus genome when infected at MOI 0.01, while only around 28% was removed when lower MOI 0.0025 was used (Figure 4E).

### 3.4. Characterization of the Recombinant DEV-HA

**Figure 5:**
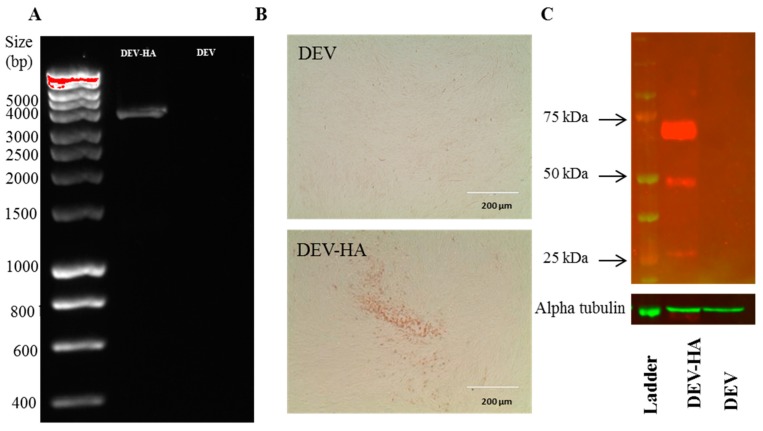
Characterization of DEV-HA. (**A**) Detection of HA cassette insertion by PCR; (**B**) detection of HA expression by immunochemistry; (**C**) detection of HA expression by Western Blot. Comparable protein loading in each lane was demonstrated by alpha tubulin detection.

Next, the insertion of HA cassette between UL26-27 was assessed using PCR. Primer DEV-UL26 and 27-F and DEV-UL26 and 27-R (Table 2) were used to amplify the intergenic region between UL26 and UL27 with HA insertion. PCR analysis showed bands of the expected size of 3900 bp (Figure 5A). The expression of HA was then determined by immunochemistry staining (IFA) (Figure 5B). As expected, cells infected with DEV-HA showed clear HA positive staining while no staining observed in cells infected with parental DEV virus. H5HA-specific chicken serum was used as a primary antibody to test HA expression by Western blot (Figure 5C). Both HA0 and the cleaved form of HA0: HA1 and HA2 were evident.

**Figure 6:**
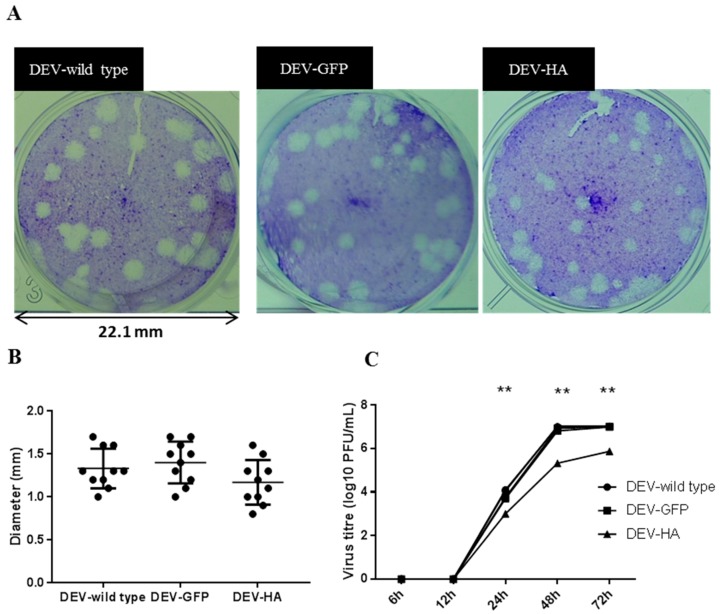
Plaque morphology and replication kinetics of wild type and recombinant DEV viruses. (**A**) Plaque morphology of DEV wild type, DEV-GFP and DEV-HA; (**B**) the plaque size of DEV wild type, DEV-GFP and DEV-HA; (**C**) multi-step growth curve of DEV wild type, DEV-GFP and DEV-HA. CEF cells were infected with DEV at MOI 0.01. Viruses were harvested at 6 h, 12 h, 24 h, 48 h and 72 h post-infection. ** *p* < 0.001.

The replication properties of the recombinant DEV-HA vaccine were assessed by virus plaque size morphology and by multi-step replication kinetics with the comparison to wildtype DEV and DEV-GFP viruses. CEF cells were infected with DEV, DEV-GFP and DEV-HA at MOI 0.01 and cells were fixed with 1% crystal violet when obvious plaque was formed. Diameter of at least 5 plaques of each virus was measured. There was no significant difference in the plaque sizes among all three viruses, though plaque size of DEV-HA appeared comparatively smaller (Figure 6A,B). Next, the multi-step replication kinetics of the three viruses was compared. DEV and DEV-GFP have similar titres (≈10^7^ PFU/mL at 72 h post-infection); however, DEV-HA vaccine virus showed significantly lower titres (≈10^6^ PFU/mL at 72 h post-infection) than both DEV-GFP and DEV wild type virus (Figure 6C), suggesting DEV-carrying HA-expressing cassette caused retardation in virus replication ability.

## 4. Discussion

To our knowledge, this is first study to explore the NHEJ-CRISPR/Cas9 system for DEV vaccine development. Here, we have demonstrated that the NHEJ-CRISPR/Cas9 system is an efficient method for gene knock-in with minimal indel generation. Furthermore, a universal donor system was established by introducing a bait sequence from the copGFP. It can be shared between different vector systems and will be beneficial in resource sharing and developing vaccines against a broad range of pathogens. In addition, the Cre-Lox system was demonstrated to be efficient for excision of the reporter gene from the virus genome in a dose-dependent manner.

CRISPR/Cas9 is an efficient tool for gene modification, and two distinct mechanisms, NHEJ and HDR, were used for recombinant vaccine development. NHEJ is error-prone and unpredictable, as the indel may be introduced during gene modification. So far, the majority of research favors the HDR mechanism due to its precision in modification [3,11,15]. However, NHEJ is generally more efficient and occurs throughout the cell cycle whereas HDR is less frequent, occurring only during S and G2 phases of the cell’s life-cycle [17]. Additionally, NHEJ is also free from the restriction of homology arms. Xu and colleagues used the NHEJ mechanism for RFP knock-in pseudorabies and reached high knock-in efficiency [18]. However, only a single bait sequence from the virus genome was introduced to the donor plasmid, which may lead to large undesirable plasmid vector segment insertion into the recombinant viruses. In our system, two duplicate bait sequences from the copGFP were introduced to the donor plasmids. A guide RNA was introduced to cut and release the insertion segment. Because the bait sequence is independent of virus and mammalian cell genomes, the donor plasmids constructed can be shared widely between different insertion sites of one virus vector or between different virus vectors.

Consistent with previous applications of reporter gene knock-in in human cells via NHEJ-CRISPR/Cas9 [15], we found minimal evidence for indels at the junction site of insertion. No vector DNA was found in the recombinant viruses. Because the indels occurred within the bait sequence, the open reading frame of the inserted expression cassette was not affected. As expected, gene expression from the knock-in cassette was not impacted by orientation of insertion.

In agreement with the study by Bi et al., we found the highest knock-in efficiency happened at 24 h rather than 6 h post-transfection [12]. This is probably linked to the peak time for the expression of heterologous genes post-plasmid transfection. As the virus dose increased the knock-in efficiency dropped, likely due to excessive quantities of viral genomes entering the cells.

GFP expression cassette knock-in between the UL26 and UL27 intergenic region did not alter plaque size and the growth kinetics of DEV. This is in line with previous studies showing that H5HA insertion did not affect the growth property of DEV [3,13]. However, plaques appeared relatively smaller and growth was markedly delayed when H5HA expression cassette was inserted. We speculate that this might be due to an influenza strain-specific effect or over expression of HA antigen. Alternatively, smaller plaque size may reflect the fact that different promoter and terminator elements were used in this study as compared to previous publications [3,13]. Potential implications of slower replication of recombinant DEV-HA on the vaccine efficiency warrant further research.

To conclude, our study demonstrated that NHEJ-CRISPR/Cas9 system together with Cre-Lox is a powerful and speedy technology in recombinant vaccine development.

## Figures and Tables

**Table 1:** The guild RNA.

sgRNA	Target Sequence 5′–3′	PAM	Gene Locus
Sg1	GGGTCCAATAACGACCGTCG	TGG	UL26-UL27
Sg2	GAGCGTATAGTTTAATCGGG	AGG	UL26-UL27
Sg3	TTTTCCACGACGGTCGTTAT	TGG	UL26-UL27
SgU	GAGATCGAGTGCCGCATCAC	CGG	copGFP

PAM: Protospacer adjacent motif.

**Table 2:** Primer list.

Primer Name	Sequence 5′–3′
DEV-HRM-F	TAAAAATTATCCCAAAGCTGTTGCG
DEV-HRM-R	CTGGCAAATATGACAACTTTAGCAA
DEV-UL26 and 27-F	GGACTTATGCTTTGTATCAAT
DEV-UL26 and 27-R	GGGACTAAATTGTTAATTGTTAC
Sense-Right-F	GGGAGGATTGGGAAGACAATAG
Sense-Right-R	TCCAGAATGTTCAAACGGAGAT
Sense-Left-F	GCTGTTGCGTCTCATTGTTG
Sense-Left-R	AAGGGCCATAACCCGTAAAG
Anti-sense-Right-F	AGCCAATTCCCACTCCTTTC
Anti-sense-Left-R	CATCGCATTGTCTGAGTAGGT
